# A Hybrid Na//K^+^-Containing Electrolyte//O_2_ Battery with High Rechargeability and Cycle Stability

**DOI:** 10.34133/2019/6180615

**Published:** 2019-01-16

**Authors:** Zhuo Zhu, Xiaomeng Shi, Dongdong Zhu, Liubin Wang, Kaixiang Lei, Fujun Li

**Affiliations:** Key Laboratory of Advanced Energy Materials Chemistry (Ministry of Education), College of Chemistry, Nankai University, Tianjin 300071, China

## Abstract

Na-O_2_ and K-O_2_ batteries have attracted extensive attention in recent years. However, the parasitic reactions involving the discharge product of NaO_2_ or K anode with electrolytes and the severe Na or K dendrites plague their rechargeability and cycle stability. Herein, we report a hybrid Na//K^+^-containing electrolyte//O_2_ battery consisting of a Na anode, 1.0 M of potassium triflate in diglyme, and a porous carbon cathode. Upon discharging, KO_2_ is preferentially produced via oxygen reduction in the cathode with Na^+^ stripped from the Na anode, and reversely, the KO_2_ is electrochemically decomposed with Na^+^ plated back onto the anode. The new reaction pathway can circumvent the parasitic reactions involving instable NaO_2_ and active K anode, and alternatively, the good stability and conductivity of KO_2_ and stable Na stripping/plating in the presence of K^+^ enable the hybrid battery to exhibit an average discharge/charge voltage gap of 0.15 V, high Coulombic efficiency of >96%, and superior cycling stability of 120 cycles. This will pave a new pathway to promote metal-air batteries.

## 1. Introduction

The Li-O_2_ batteries have attracted a lot of academic research interests in recent years [1, 2]. However, the electrochemical decomposition of the discharge product of Li_2_O_2_ based on the two-electron reaction of O_2_^2−^ → O_2_ is difficult and leads to a large discharge/charge voltage hysteresis of ~1.0 V [3–9]. Alternatively, with one-electron transfer the redox couple of O_2_/O_2_^−^ is highly reversible, but the O_2_^−^ in the form of LiO_2_ is metastable and can only be detected at the beginning of a discharging process of Li-O_2_ batteries [10–12]. In the presence of Na^+^ or K^+^, the O_2_^−^ generated in oxygen reduction reaction (ORR) can be captured to form NaO_2_ and KO_2_ as the main discharge product of Na-O_2_ and K-O_2_ battery, respectively [13–15]. This allows their decomposition with low charge overpotential of ~0.2 V. Therefore, construction and understanding of metal-oxygen batteries based on one-electron transfer of O_2_/O_2_^−^ will be intrinsically important to improve rechargeability and cyclability.

Na-O_2_ and K-O_2_ batteries are based on the reversible one-electron reaction of O_2_/O_2_^−^ at cathodes and the stripping/plating of Na or K at anodes [15–17]. Chemical nature of the intermediate products dictates their electrochemical performance. In Na-O_2_ battery, the discharge product NaO_2_ was soluble and active, and the liberated O_2_^−^ partially reacted with the electrolyte solvent or water to produce Na_2_O_2_·H_2_O [17, 18]. NaO_2_ was reported to be converted to Na_2_O_2_ as the final product [19, 20]. The presence of Na_2_O_2_ or Na_2_O_2_·H_2_O in cathodes results in high charge overpotentials and low Coulombic efficiency, which triggers parasitic reactions and hence poor cycle life [17, 21]. Comparatively, KO_2_ is thermodynamically stable in K-O_2_ battery: when it was submerged in a dimethoxyethane (DME) based electrolyte, no byproducts were detected even for one month and a high Coulombic efficiency of 98% was achieved in a discharge/charge cycle [22]. However, the K anode is active to both glyme molecules and dissolved O_2_ in electrolytes, inducing high overvoltages in K-O_2_ batteries [23]. These necessitate intrinsic variation in the reaction pathways of either Na-O_2_ or K-O_2_ batteries for low overvoltages and improved cycle performance by mitigating the active discharge product of NaO_2_ or anode of K, and the detrimental dendrites of Na or K [22–26].

**Figure 1:**
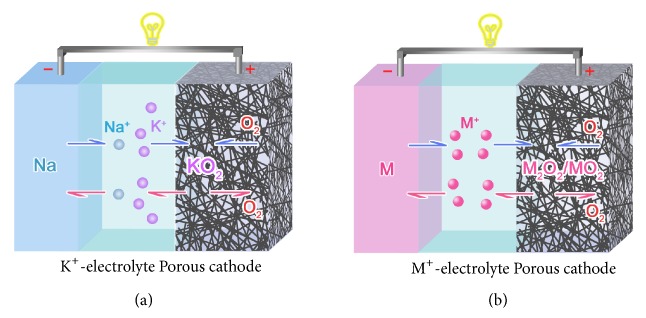
**Schematic illustration of the NKO and conventional metal-O**_2_ **battery**. (a) NKO battery consisted of a Na anode, glassy fiber separator impregnated with 1.0 M of KOTF in G2, and a porous SP cathode. (b) Conventional metal-O_2_ battery adopting an electrolyte containing the same element M^+^ as the anode (M = Na/K).

Herein, we report a hybrid Na//K^+^-containing electrolyte//O_2_ (NKO) battery, which consists of a Na anode, 1.0 M of potassium triflate (KOTF) in diglyme (G2), and porous Super P (SP) carbon cathode, as comparatively shown with the configuration of conventional metal-O_2_ battery in Figure 1. In a discharging process, O_2_ is reduced in cathode to preferentially combine with K^+^ in electrolyte to form KO_2_, and Na^+^ is stripped from anode; in a reverse process, KO_2_ is electrochemically decomposed to K^+^ and O_2_ in cathode, and Na^+^ is reversibly plated back onto anode. The new reaction pathway in the NKO battery, which is different from either Na-O_2_ or K-O_2_ battery, can effectively circumvent the reactivity of NaO_2_ and K anode with electrolyte, and the K^+^ in the electrolyte promotes uniform plating of Na in the charging processes. These guarantee the NKO battery to exhibit a low average discharge/charge voltage gap of 0.15 V and high Coulombic efficiency of >96% after 100 cycles. This work provides an intrinsically new strategy for high-performance metal-oxygen batteries.

## 2. Results

### 2.1. Chemistry of NKO Battery

**Figure 2:**
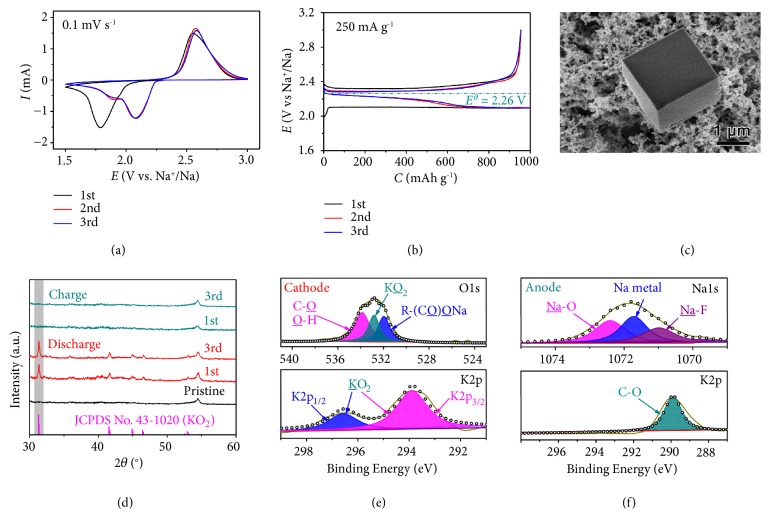
**Electrochemical measurements and characterization of the NKO battery.** (a) CV curves at 0.1 mV s^−1^ from 1.5 to 3.0 V. (b) Discharge/charge profiles of the initial three cycles at 250 mA g^−1^. (c) SEM image of the discharged SP cathode. (d) XRD patterns of the discharged and charged SP cathodes. (e, f) XPS spectra of the discharged SP cathode and the charged Na anode, respectively.

The configuration of hybrid NKO battery is depicted in comparison with the conventional metal-O_2_ battery in Figures 1(a) and 1(b), respectively. The NKO battery consists of a Na anode, 1.0 M of KOTF in G2 impregnated in glassy fiber separator, and a porous SP cathode, which is different from the conventional metal-O_2_ battery in the electrolyte composition. The cyclic voltammetry (CV) curves of the NKO battery over the initial three cycles at a scan rate of 0.1 mV s^−1^ are shown in Figure 2(a). It is clear that the broad peak in the cathodic scan is attributed to oxygen reduction and the current peak in the reverse scan represents oxygen evolution. In the following cycles, the reduction peak is positively shifted to 2.08 V as well as a shoulder one at 1.90 V, and an oxidation peak is observed at 2.57 V. Overlapping of these current peaks suggests good reversibility. The discharge/charge curves of the NKO battery in Figure 2(b) present two discharge plateaus beyond the first cycle and one charge plateau, in agreement with the CV curves. It can be found that the average discharge/charge voltage gap is as low as 0.15 V at 250 mA g^−1^, which is smaller than those of Na-O_2_ and K-O_2_ batteries in [Supplementary-material supplementary-material-1].

The discharge product of the NKO battery is shown to exhibit as a cube on the SP cathode in the scanning electron microscope (SEM) image of Figure 2(c). It is identified as pure KO_2_ by X-ray diffraction (XRD) in Figure 2(d), which is matching well with the diffraction peaks of the standard (No. 43-1020). Raman spectra in [Supplementary-material supplementary-material-1] also confirm the formation of high-purity KO_2_ at the discharged SP cathode, which presents a characteristic peak at 1142 cm^−1^ with the other two broad peaks of G band (1582 cm^−1^) and D band (1350 cm^−1^) of the SP carbon black [15, 27, 28]. X-ray photoelectron spectroscopy (XPS) is employed to analyze the discharged products on the SP cathode. As shown in Figure 2(e) and [Supplementary-material supplementary-material-1], the XPS spectra of O1s, K2p, C1s, and Na1s of discharged SP cathode are comparatively displayed. The R-COONa, C-O-C, and C-C with the characteristic peaks at 290.2, 287.8, and 284.6 eV in the C1s spectra and R-COONa at 1072.8 eV in the Na1s spectra are stemmed from the binder of CMC. The KO_2_ is further evidenced by the O1s signal at 533.2 eV and K2p signal at 294.1 eV in Figure 2(e) of XPS [29, 30]. After charging, the typical diffraction peaks and Raman signals of KO_2_ in Figure 2(e) and [Supplementary-material supplementary-material-1] disappear, indicating reversible decomposition of KO_2_.

To quantify the KO_2_ generated in a discharging process, iodometric titration is performed on the discharged SP cathode (Supplementary Methods). The discharge product of KO_2_ reacts with H_2_O via 2KO_2_(s) + 2H_2_O(l) → H_2_O_2_(l) + 2KOH(aq.) + O_2_(g), then H_2_O_2_ oxidizes iodide to iodine, which is titrated by Na_2_S_2_O_3_ ([Supplementary-material supplementary-material-1]; titration processes in Supplementary Methods) [31–33]. By comparing the electrons contributing to the discharge capacity to the O_2_ derived from the discharge product of KO_2_ via titration, the ORR in a discharging process involves 1.01e^−^ per O_2_ molecule. These reveal the highly reversible one-electron transfer process and negligible parasitic side reaction in the NKO battery.

On the Na anode, in either a discharging or charging process of the NKO battery in Figure 2(f) or [Supplementary-material supplementary-material-1], the derived species on the Na surface are almost identical. The XPS spectrum of Na1s in Figure 2(f) can be deconvoluted and assigned to the Na-O species, Na, and NaF at 1072.6, 1071.6, and 1071 eV, respectively [34]. Of note, there are no signals related to the K species in the K2p spectra on both the charged and discharged Na anode of the NKO battery in Figure 2(f) and [Supplementary-material supplementary-material-1], respectively, and the only XPS peak at 289.8 eV is attributed to the C-O species. This indicates that no K stripping and platting occur on the Na anode in a cycle; that is, in a discharging process of the NKO battery Na^+^ is generated from the Na anode into the electrolyte and in a reverse process Na^+^ is preferentially plated back onto the Na anode, rather than K^+^.

### 2.2. Reaction Mechanism

**Figure 3:**
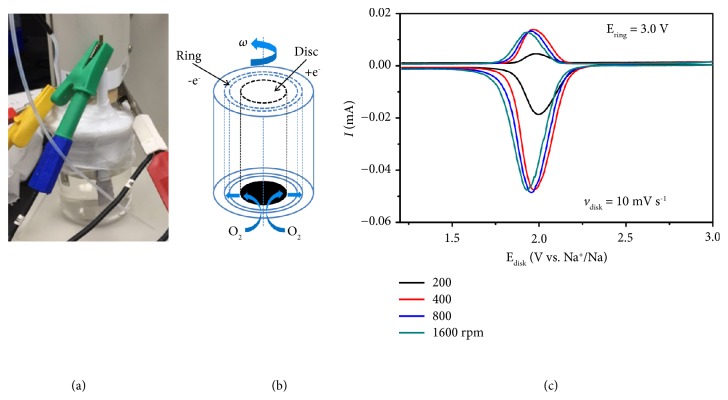
**Electrochemical measurements and characterization**. (a) Photograph of RRDE in a three-electrode cell. (b) Schemes of RRDE. (c) Current responses of oxygen reduction/evolution reaction on RRDE.

Rotating ring-disk electrode (RRDE) in a three-electrode cell is employed to study the reaction process in Figure 3(a). The linear sweep voltammetry (LSV) reveals one disk current response associated with reduction of oxygen to superoxide and one significant ring current for oxidation of superoxide, respectively, in Figures 3(b) and 3(c). It confirms a one-electron and single-step oxygen reduction/evolution process in the NKO battery [35, 36], which is in accordance with the iodometric titration. Although there are two reduction peaks and discharge plateaus in the CV curves and discharge/charge profiles in Figures 2(a) and 2(b), respectively, only KO_2_ is detected as the discharge product.

In [Supplementary-material supplementary-material-1], the NKO battery is controlled to discharge for limited capacities of 250, 500, and 1000 mAh g^−1^, respectively, and two discharge plateaus remain, regardless of the discharge depth. XRD patterns in [Supplementary-material supplementary-material-1] display the characteristic diffraction peaks of KO_2_, and no other products are detected. It further confirms the discharge product of KO_2_ in the NKO battery. On the other hand, a symmetric Na/Na cell with 1.0 M KOTF in G2 is constructed and tested in O_2_ atmosphere. Its voltage profiles are shown in [Supplementary-material supplementary-material-1], and there exist two symmetric flat plateaus except for the first cycle. For a comparison, with 1.0 M NaOTF in G2 the symmetric Na/Na cell shows only one plateau in [Supplementary-material supplementary-material-1]. These are correlated to the interfacial charge transfer between the Na anode and the K^+^- or Na^+^-containing electrolyte and are consistent with the discharge/charge profiles of the NKO and Na-O_2_ batteries in [Supplementary-material supplementary-material-1]. Therefore, it is believed that the two reduction peaks in CVs and two discharge plateaus of the NKO battery are stemmed from the interfacial polarization of Na anode/K^+^ in electrolyte.

**Figure 4:**
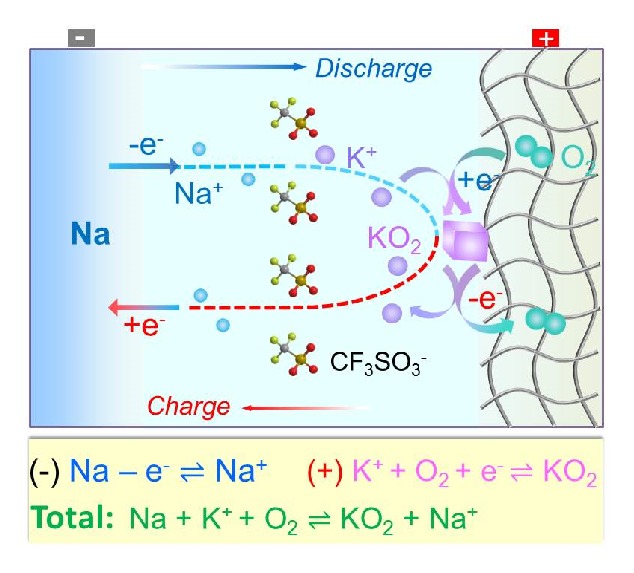
Reaction pathways of the NKO battery in a discharge and charge cycle.

The reaction mechanism of the NKO battery is conclusively described in Figure 4. In a discharging process, O_2_ enters the porous SP cathode and is reduced to O_2_^−^, and it then preferentially combines with K^+^ in the electrolyte to form solid KO_2_; simultaneously, Na^+^ is produced from the Na anode into the electrolyte for charge compensation. In a following charging process, the KO_2_ is electrochemically oxidized to release K^+^ into the electrolyte and O_2_ on the cathode; at the same time, Na^+^ accepts one electron to be plated back onto the Na anode because of the chemical activity order of Na < K, leaving K^+^ in the electrolyte.

Precisely, electrochemical reactions are governed by the redox potentials, which are related to the concentrations of active species according to Nernst equation. Based on the estimation in Supplementary Methods, KO_2_ is preferentially produced when the concentration ratio of [K^+^]:[Na^+^] is higher than 3.2:1. To deliberately tune the discharge product of the NKO battery with a discharge limit of 1000 mAh g^−1^, electrolytes with different ratios of [K^+^]:[Na^+^] are applied, as shown in [Supplementary-material supplementary-material-1]. When the initial ratio of [K^+^]:[Na^+^] is larger than 0.75:0.25, the discharge product is pure KO_2_, and when less than this critical point, it is a mixture of KO_2_ and NaO_2_, which are identified by both XRD and Raman spectra in [Supplementary-material supplementary-material-1]. In a discharging process, [K^+^] in the electrolyte is decreasing for formation of KO_2_ with increasing of [Na^+^] from the Na anode, leading to changes of [K^+^]:[Na^+^]. Hence, KO_2_ and NaO_2_ coexist as the discharge product when the capacity limit is changed from 500 and 1000 mAh g^−1^ to 2000 or 4000 mAh g^−1^, as confirmed in [Supplementary-material supplementary-material-1]. Similarly, K^+^ can be plated together with Na^+^ when the [K^+^]:[Na^+^] is higher than 11368 (see the estimation process in Supplementary Methods). It is revealed that the relative amount of K^+^ in the electrolyte and the discharge capacity are crucial for construction of the NKO battery.

### 2.3. Electrochemical Performance

**Figure 5:**
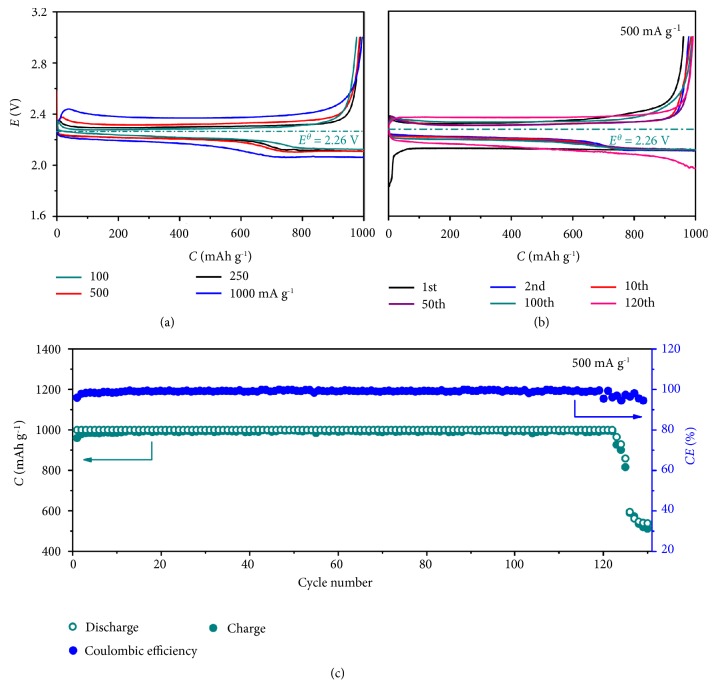
**Electrochemical performance of the NKO battery.** (a) Discharge/charge profiles of the tenth cycle at varied current densities. (b) Discharge/charge profiles of the selected runs over 100 cycles at 500 mA g^−1^. (c) Cycling stability.

Symmetric Na/Na cells were used to evaluate interfacial stability during Na plating/stripping. With 1.0 M of NaOTF in G2 as electrolyte, the symmetric cell only works for 43 hrs in O_2_ atmosphere because of short circuit in [Supplementary-material supplementary-material-1], while it presents a stable voltage profile for 180 hrs in 1.0 M of KOTF in G2 in O_2_ atmosphere in [Supplementary-material supplementary-material-1], indicating suppression of Na dendrites in the presence of K^+^ in electrolyte on sodium plating/stripping. On the other hand, theoretical equilibrium voltage (*E*^*θ*^) of the NKO battery is 2.26 V.  Figure 5(a) shows the discharge/charge profiles of the NKO battery at current densities from 100 mA g^−1^ to 250, 500, and 1000 mA g^−1^. At 100 mA g^−1^, the overvoltages in the discharging and charging process are 0.03 and 0.12 V for the two respect discharge plateaus, and 0.02 V, respectively. They result in small discharge/charge overvoltage gaps of 0.05 and 0.14 V for the two plateaus. The charge overvoltage of as low as 0.03 V is much smaller than that in Na-O_2_ or K-O_2_ battery in [Supplementary-material supplementary-material-1] and the reported Li-O_2_ batteries [37–42]. Even when the current density is increased by two and four times, very small discharge/charge overvoltage increase is observed in Figure 5(a). It is attributed to the high conductivity of the K^+^-containing electrolyte ([Supplementary-material supplementary-material-1]) and discharge product KO_2_ ([Supplementary-material supplementary-material-1]) [15], and the Na anode of the unique NKO battery.

The NKO battery is continuously discharged and charged at 500 mA g^−1^ for 120 cycles, and the selected cycles are displayed in Figure 5(b). The discharge and charge curves are almost overlapped except the first cycle, indicating good rechargeability and cycle stability of the NKO battery. The discharge and charge capacities in the 120 cycles are almost constant, and the corresponding Coulombic efficiency in each run is approaching 99% in Figure 5(c) with an initial Coulombic efficiency of high up to 96.5%. The discharge/charge overvoltage gaps become larger after 120 cycles, which may be ascribed to the evaporation of electrolyte and the increased resistance of the SP cathode. In contrast, the Na-O_2_ and K-O_2_ batteries are cycled at the same current of 500 mA g^−1^ in [Supplementary-material supplementary-material-1], in which both of them can only run for tens of cycles with obviously increasing discharge and charge overvoltages. The demonstrated small discharge/charge voltage gap, good rechargeability, and long cycle stability of the NKO battery are benefited from the good stability and conductivity of KO_2_ and the Na anode in place of K.

### 2.4. Analyses on Cycled Electrodes

XRD patterns of the discharged/charged SP cathodes during 100 cycles are displayed in [Supplementary-material supplementary-material-1]. It is clear that the only product of KO_2_ is reversibly generated and decomposed during the cycles, evidenced by the appearance and disappearance of its characteristic diffraction peaks. Raman spectra of the discharged SP cathodes in [Supplementary-material supplementary-material-1] also confirm the production of KO_2_ by its typical O-O^−^ Raman band at 1142 cm^−1^ and no formation of NaO_2_ during 100 cycles. After recharge, the discharged product KO_2_ is decomposed with disappearance of its characteristic Raman band in [Supplementary-material supplementary-material-1]. The reversible formation and decomposition of KO_2_ during the cycles are related to the component stability of the NKO battery and consistent with the discharge/charge profiles and cycle stability in Figure 5.

**Figure 6:**
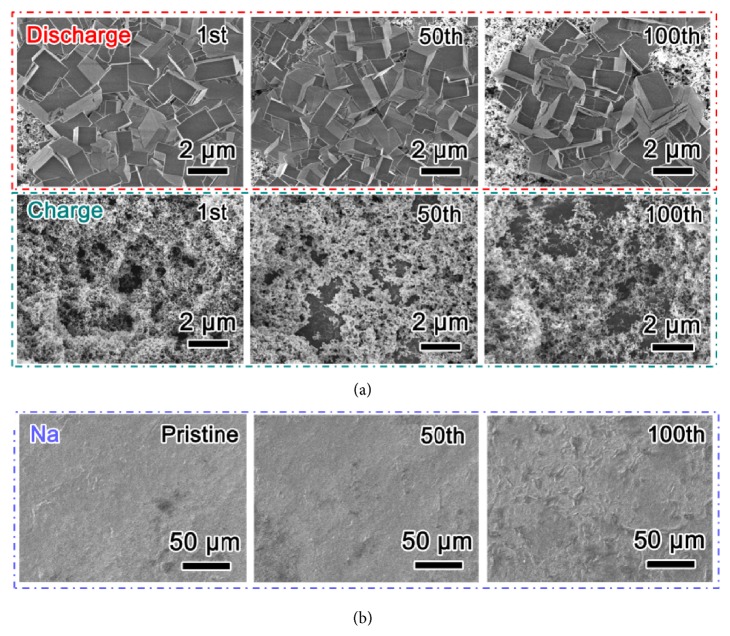
**SEM images of the discharged/charged SP cathodes and Na anodes of the NKO battery. **(a) SEM images of the selected runs of discharged/charged cathodes. (b) SEM images of the charged Na anodes during 100 cycles.

SEM is further employed to monitor the morphologies of KO_2_ during 100 cycles, as shown in Figure 6(a). The porous nature of the fresh SP cathode composed of aggregates of SP nanoparticles is revealed in [Supplementary-material supplementary-material-1]. In all the discharged SP cathodes, the product of KO_2_ is visible and in shape of cube, which is in good agreement with the previous reports [23, 43]. The particle size is estimated to be 1-3 *μ*m, but such big size does not induce as large charge overvoltage as Li-O_2_ battery because of its good conductivity [22, 44]. After recharge, the KO_2_ is decomposed and the SP cathode becomes porous during cycles, which indicates formation of KO_2_ both on and beneath the electrode surface. In addition, the Na anode of the NKO battery during 100 cycles is shown in Figure 6(b), as well as the Na anode of Na-O_2_ battery in [Supplementary-material supplementary-material-1]. It is apparent that the Na surface of NKO battery is smooth during cycles, while the Na surface of Na-O_2_ battery is rough with Na particles of twenties of micrometer in size. The K^+^ in the electrolyte promotes the uniform stripping/plating of Na^+^ in charging processes via a self-healing electrostatic shield mechanism [45, 46]. This is linked to the difference in cycle stability of the NKO and Na-O_2_ battery.

## 3. Discussion

A novel NKO battery is successfully constructed with a Na anode, 1.0 M of KOTF in G2, and SP cathode. In a discharging process, KO_2_ is preferentially produced on the cathode via ORR involving one-electron transfer, and Na^+^ is stripped from the Na anode into electrolyte, and in a charging process, the KO_2_ is electrochemically decomposed to K^+^ and O_2_, and Na^+^ is plated back onto the anode. This new battery configuration effectively circumvents the usage of K to avoid its reaction with electrolytes and formation of instable NaO_2_ as discharge product. Favored by the good stability and conductivity of KO_2_ and the stable Na stripping/plating in the presence of K^+^, the NKO battery exhibits an ultralow charge overvoltage of 0.03 V, a small average discharge/charge voltage gap of 0.15 V, high Coulombic efficiency of >96%, and cycle life of 120 cycles. The exciting battery performance will shed light on design of batteries with high rechargeability and good cycle stability and promote the development of metal-air batteries.

## 4. Materials and Methods

### 4.1. Battery Assembly

Super P (SP) and sodium carboxymethylcellulose (CMC) (90:10 by weight) were mixed in an ethanol aqueous solution, and the resulting slurry was coated onto a carbon paper (TGP-H-060 carbon paper, Torray) with a carbon loading of 0.4 ± 0.1 mg cm^−2^. The coated carbon paper was dried at 80°C for 12h under vacuum. It was then punched to electrode pellets of 10 mm in diameter. The NKO battery was assembled in CR2032 coin cells in an argon-filled glovebox with water and O_2_ content both less than 0.1 ppm. It consisted of a Na foil as anode (12 mm in diameter), glassy fiber separators (16 mm in diameter) impregnated with 200 *μ*L of electrolyte, and carbon cathode. The electrolyte was prepared by dissolving potassium triflate (KOTF) into distilled G2 in the Ar-filled glovebox with a concentration of 1.0 M. The mixed electrolyte was composed of KOTF and sodium triflate (NaOTF) in G2, and the total concentration remained 1.0 M. For Na-O_2_ and K-O_2_ battery, the preparation processes were the same except the applied salts. The symmetric Na/Na cell was composed of two Na foils separated by a glassy fiber separator incorporated with 1.0 M NaOTF or KOTF in G2.

### 4.2. Electrochemical Measurements

Discharge/charge tests were carried out on Land CT2001A battery instruments. Cyclic voltammetry (CV) and electrochemical impedance spectroscopy (EIS) were performed on an electrochemical workstation of Solartron 1470E. The EIS was obtained with an alternating current (ac) perturbation amplitude of 10 mV and frequencies from 100 mHz to 10 kHz. The metal-oxygen batteries were stored in an O_2_-filled glass chamber. Rotating ring-disk electrode (RRDE) measurements were performed in an O_2_-saturated electrolyte solution of 1.0 M KOTF in G2 by applying the disk voltage between 1.2 and 3.0 V at 10 mV s^−1^ and the ring voltage constant at 3.0 V against a counter electrode of Pt wire and reference electrode of Na foil. All the electrochemical measurements were performed at room temperature.

### 4.3. Characterization

Scanning electron microscopy (SEM, JEOL-JSM7500F) was employed to observe the morphologies of the electrode changes during cycles. The samples were carefully protected from exposure to air by applying conductive tape in the argon-filled glovebox during transfer to the SEM chamber. X-ray diffraction (XRD, Rigaku MiniFlex600 X-ray generator, Cu K*α* radiation, *λ* = 1.5406 Å) and Raman spectroscopy (Thermo Fisher Scientific with excitation at 532 nm) were applied to identify the discharge/charge products on the cathodes during cycles. X-ray photoelectron spectroscopy (XPS) measurements on the washed cathodes and anodes were conducted on a Perkin Elmer PHI 1600 ESCA system to analyze the surface components of the cathode and anode. For these tests, the discharged/charged electrodes were washed with dehydrated dimethoxyethane (DME, dried by 4 Å molecular sieves) and dried under vacuum to remove the residual solvents.

## Data Availability

All data needed to evaluate the conclusions in the paper are present in the paper and/or the Supplementary Materials. Additional data related to this paper may be requested from the authors.

## Supplementary Materials

Supplementary Materials Figure S1: discharge/charge profiles of NKO, Na-O_2_, and K-O_2_ battery. Figure S2: Raman spectra of the discharged and charged SP cathodes. Figure S3: XPS spectra of the discharged SP cathode in the NKO battery. Figure S4: color changes in the iodometric titration process. Figure S5: XPS spectra of the discharged Na anode in the NKO battery. Figure S6: electrochemical measurements and characterization. Figure S7: voltage profiles of the Na/Na symmetric cells with 1.0 M KOTF and NaOTF in G2, at 0.1 mA cm^−2^ in O_2_ atmosphere. Figure S8: characterization of the discharged SP cathode of the NKO battery. Figure S9: Raman spectra of the discharged SP cathodes in the NKO battery at different discharge depths. Figure S10: plots of voltage profiles versus time of the Na/Na symmetric cells with 1.0 M NaOTF and KOTF in G2, at 0.2 mA cm^−2^ in O_2_ atmosphere. Figure S11: electrochemical impedance spectroscopy of the two kinds of electrolytes, 1.0 M NaOTF and 1.0 M KOTF in G2. Figure S12: electrochemical performances of NKO, Na-O_2_, and K-O_2_ battery. Figure S13: analyses on the discharged/charged SP cathodes of the NKO battery during cycles. Figure S14: SEM images of the pristine and discharged/charged SP cathodes of the NKO battery. Figure S15: SEM images of Na anodes. Table S1: comparison of NaO_2_ and KO_2_. Supplementary Methods: the methods consist of the following: (1) iodometric titration process (preparation of standard sodium thiosulfate and titration of KO_2_ in a discharged cathode), (2) estimation of reactions occurring on the anode and cathode, and (3) calculation of theoretical equilibrium potential.
