# Supplementary material for: A Hybrid Na//K+-Containing Electrolyte//O2 Battery with High Rechargeability and Cycle Stability
Source: Research (Wash D C). 2019 Jan 16;2019:6180615. doi: 10.34133/2019/6180615 (PMC6750056; doi:10.34133/2019/6180615)
Supplement: Supplementary Materials — Figure S1: discharge/charge profiles of NKO, Na-O2, and K-O2 battery. Figure S2: Raman spectra of the discharged and charged SP cathodes. Figure S3: XPS spectra of the discharged SP cathode in the NKO battery. Figure S4: color changes in the iodometric titration process. Figure S5: XPS spectra of the discharged Na anode in the NKO battery. Figure S6: electrochemical measurements and characterization. Figure S7: voltage profiles of the Na/Na symmetric cells with 1.0 M KOTF and NaOTF in G2, at 0.1 mA cm−2 in O2 atmosphere. Figure S8: characterization of the discharged SP cathode of the NKO battery. Figure S9: Raman spectra of the discharged SP cathodes in the NKO battery at different discharge depths. Figure S10: plots of voltage profiles versus time of the Na/Na symmetric cells with 1.0 M NaOTF and KOTF in G2, at 0.2 mA cm−2 in O2 atmosphere. Figure S11: electrochemical impedance spectroscopy of the two kinds of electrolytes, 1.0 M NaOTF and 1.0 M KOTF in G2. Figure S12: electrochemical performances of NKO, Na-O2, and K-O2 battery. Figure S13: analyses on the discharged/charged SP cathodes of the NKO battery during cycles. Figure S14: SEM images of the pristine and discharged/charged SP cathodes of the NKO battery. Figure S15: SEM images of Na anodes. Table S1: comparison of NaO2 and KO2. Supplementary Methods: the methods consist of the following: (1) iodometric titration process (preparation of standard sodium thiosulfate and titration of KO2 in a discharged cathode), (2) estimation of reactions occurring on the anode and cathode, and (3) calculation of theoretical equilibrium potential. [file 6180615.f1.docx]

Supplementary Materials for

A Hybrid Na//K^+^-Containing Electrolyte//O_2_ Battery with High Rechargeability and Cycle Stability

Zhuo Zhu, Xiaomeng Shi, Dongdong Zhu, Liubin Wang, Kaixiang Lei, and Fujun Li*

**This PDF file includes:**

Fig. S1. Discharge/charge profiles of NKO, Na-O_2_, and K-O_2_ battery.

Fig. S2. Raman spectra of the discharged and charged SP cathodes.

Fig. S3. XPS spectra of the discharged SP cathode in the NKO battery.

Fig. S4. Color changes in the iodometric titration process.

Fig. S5. XPS spectra of the discharged Na anode in the NKO battery.

Fig. S6. Electrochemical measurements and characterization.

Fig. S7. Voltage profiles of the Na/Na symmetric cells with 1.0 M KOTF and NaOTF in G2, at 0.1 mA cm^-2^ in O_2_ atmosphere.

Fig. S8. Characterization of the discharged SP cathode of the NKO battery.

Fig. S9. Raman spectra of the discharged SP cathodes in the NKO battery at different discharge depths.

Fig. S10. Plots of voltage profiles versus time of the Na/Na symmetric cells with 1.0 M NaOTF and KOTF in G2, at 0.2 mA cm^-2^ in O_2_ atmosphere.

Fig. S11. Electrochemical impedance spectroscopy of the two kinds of electrolytes, 1.0 M NaOTF and 1.0 M KOTF in G2.

Fig. S12. Electrochemical performances of NKO, Na-O_2_, and K-O_2_ battery.

Fig. S13. Analyses on the discharged/charged SP cathodes of the NKO battery during cycles.

Fig. S14. SEM images of the pristine and discharged/charged SP cathodes of the NKO battery.

Fig. S15. SEM images of Na anodes.

Table S1. Comparison of NaO_2_ and KO_2_.

Supplementary Methods

**
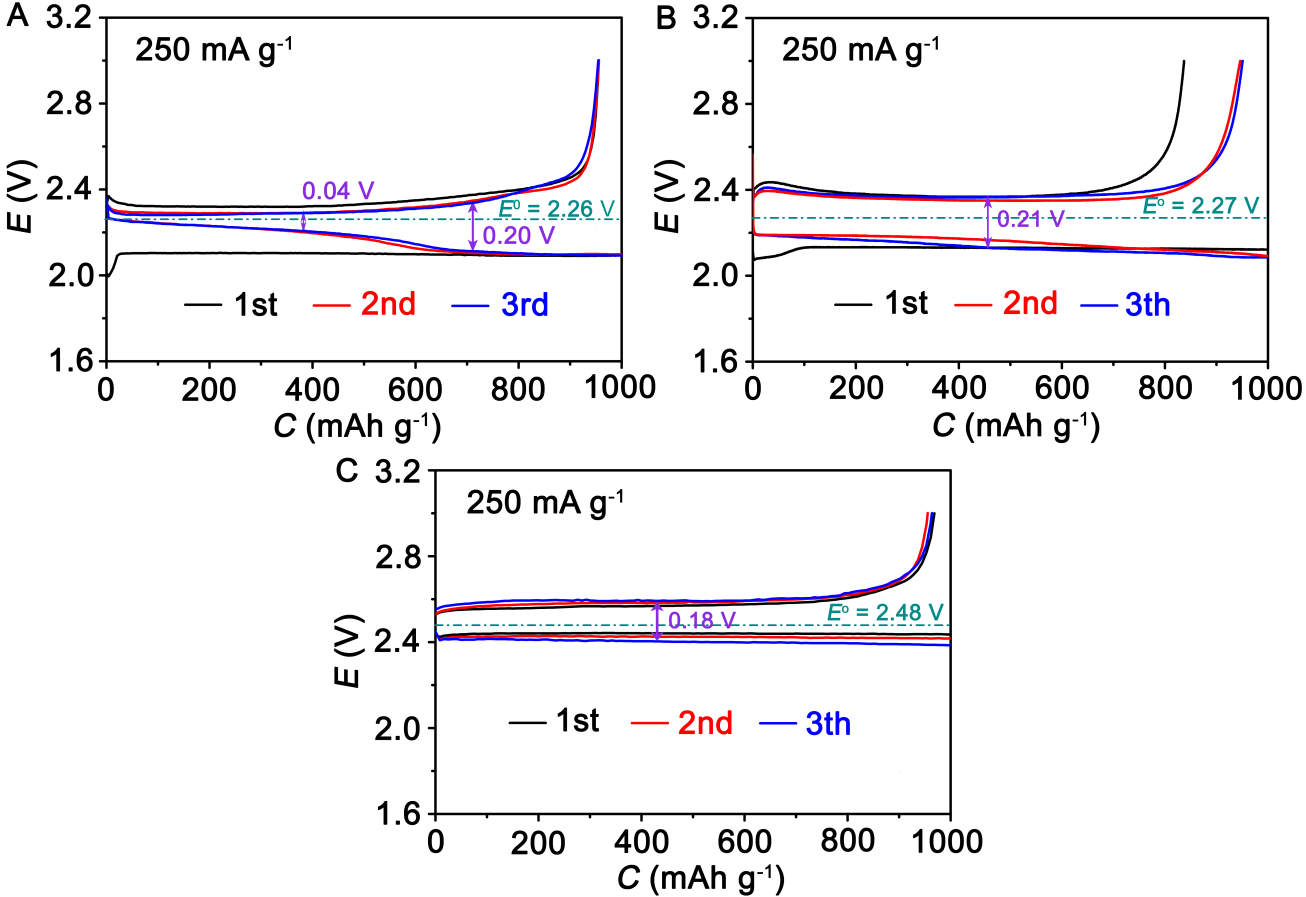
**

**Fig. S1. Discharge/charge profiles of NKO, Na-O_2_, and K-O_2_ battery.** (A) NKO. (B) Na-O_2_. (C) K-O_2_ battery at 250 mA g^-1^ with a capacity limit of 1000 mAh g^-1^.

**
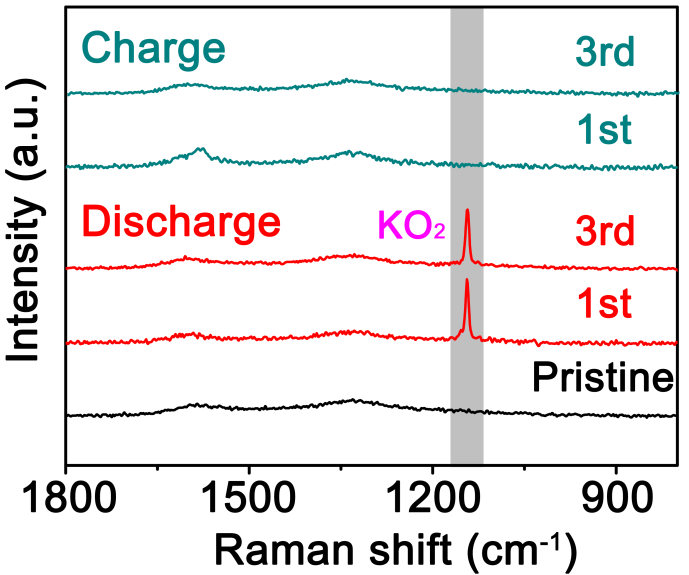
**

**Fig. S2. Raman spectra of the discharged and charged SP cathodes.**


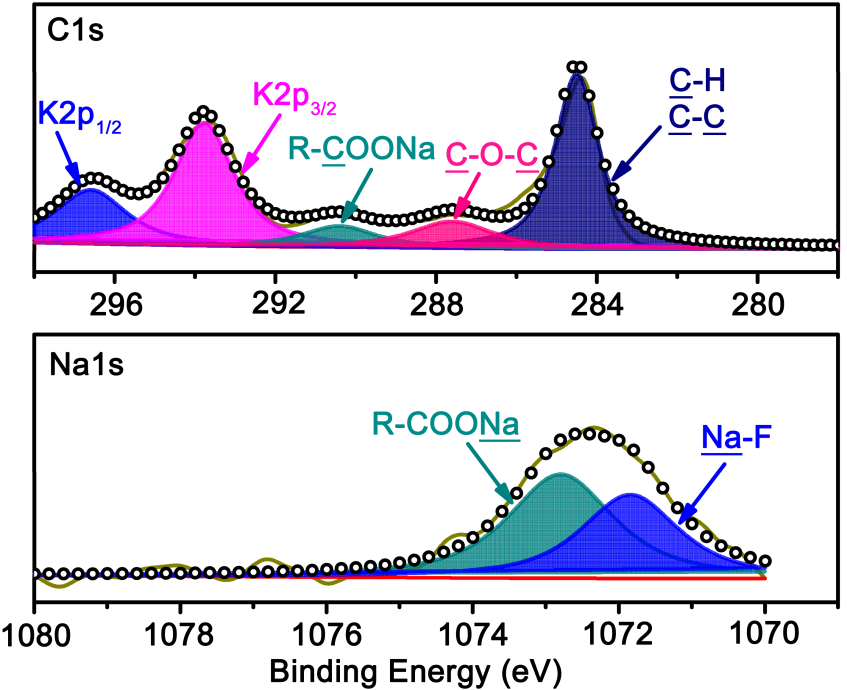


**Fig. S3. XPS spectra of the discharged SP cathode in the NKO battery.** Capacity limit: 1000 mAh g^-1^; Electrolyte: 1.0 M KOTF in G2.


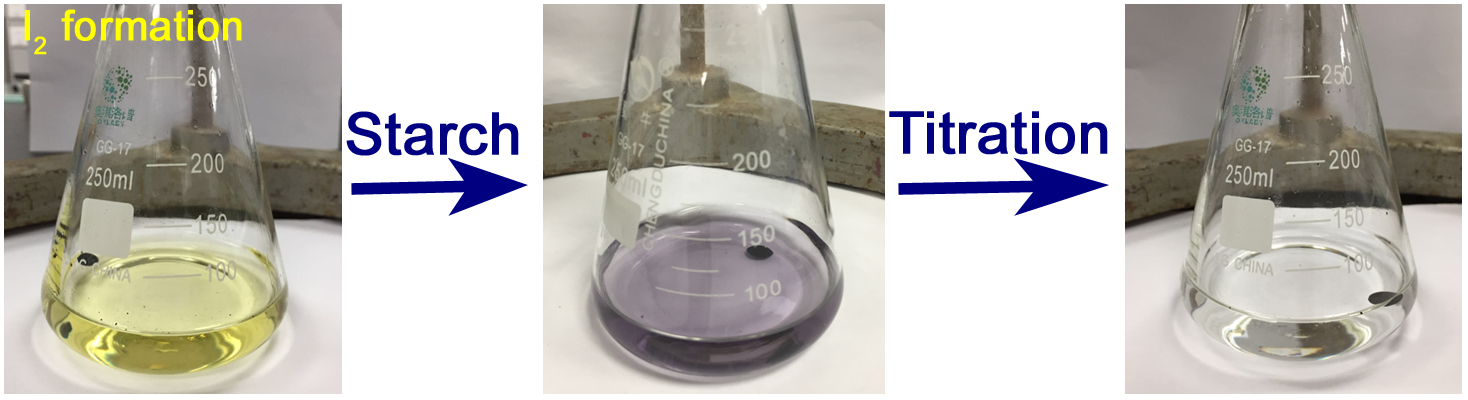


**Fig. S4. Color changes in the iodometric titration process.**


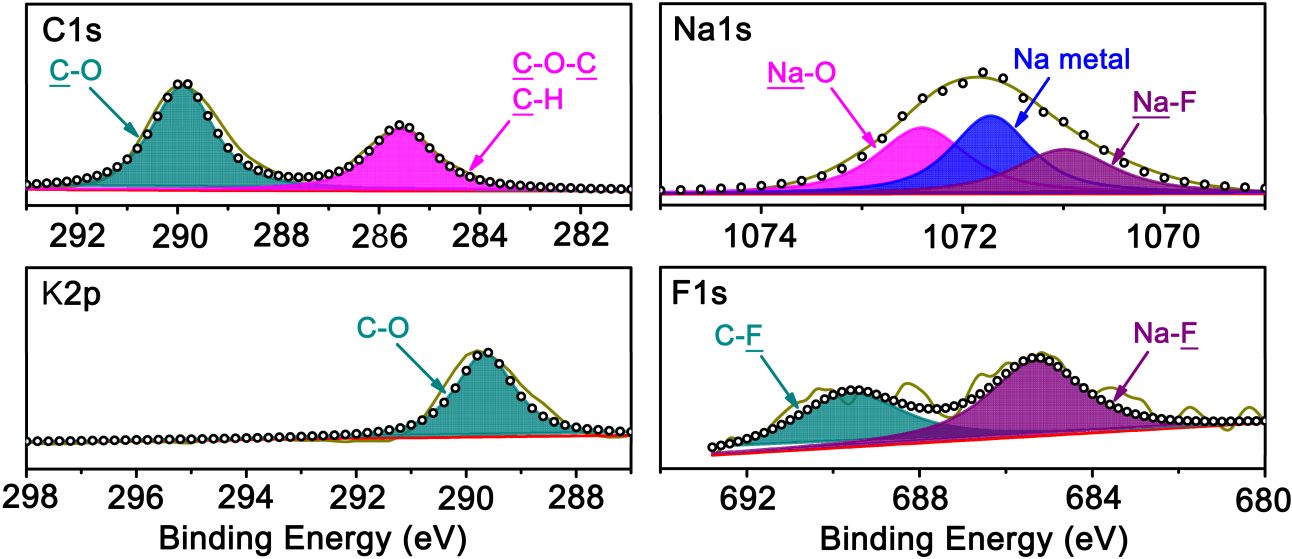


**Fig. S5. XPS spectra of the discharged Na anode in the NKO battery.** Capacity limit: 1000 mAh g^-1^; Electrolyte: 1.0 M KOTF in G2.


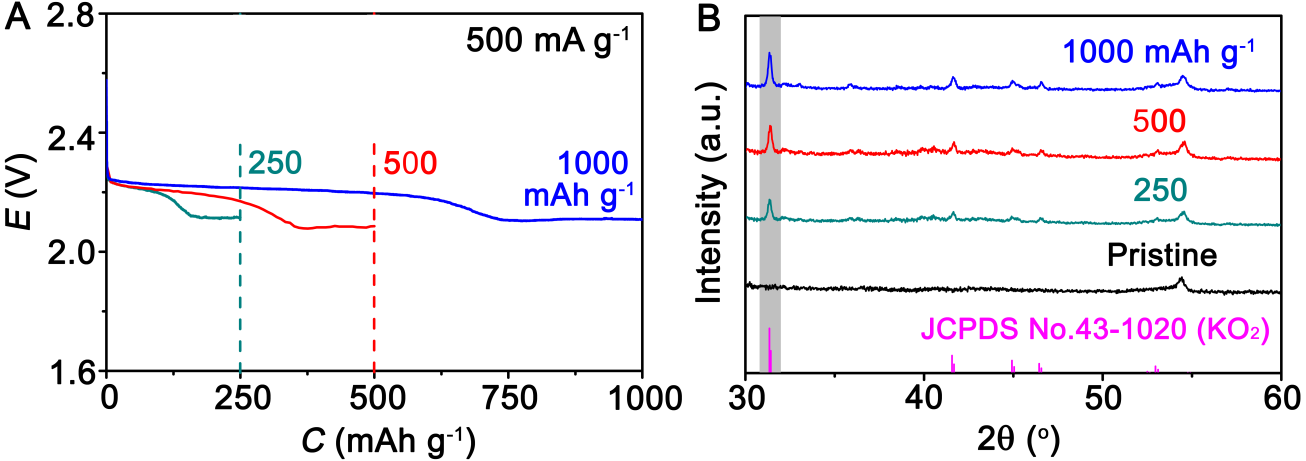


**Fig. S6. Electrochemical measurements and characterization**. (A,B) Discharge profiles of the NKO battery with varied capacity limits of 250, 500, and 1000 mAh g^-1^ at 500 mA g^-1^, and the corresponding XRD patterns of the discharged SP cathodes.


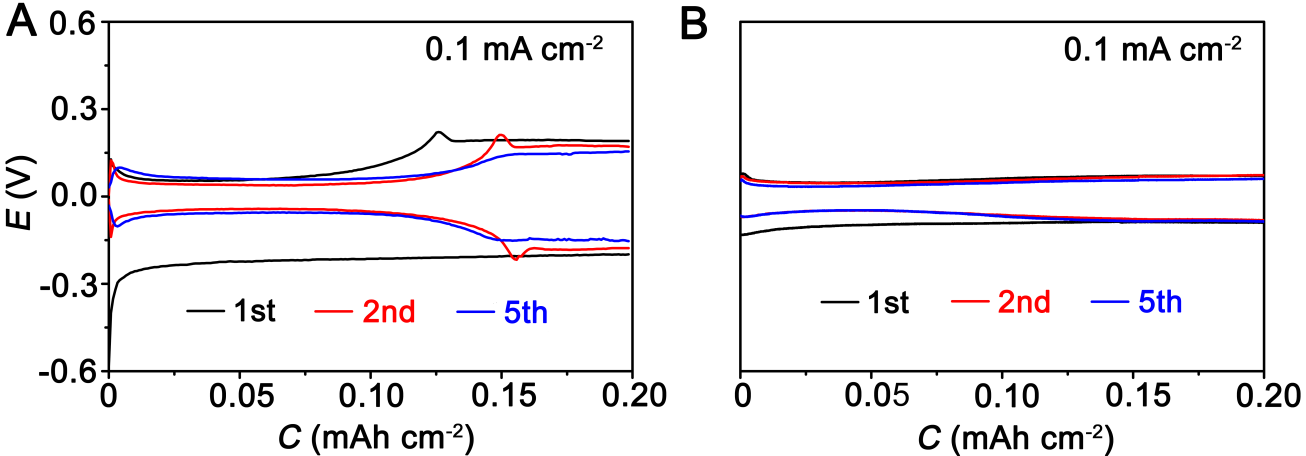


**Fig. S7.** Voltage profiles of the Na/Na symmetric cells with 1.0 M KOTF (A) and NaOTF (B) in G2, respectively, at 0.1 mA cm^-2^ in O_2_ atmosphere.


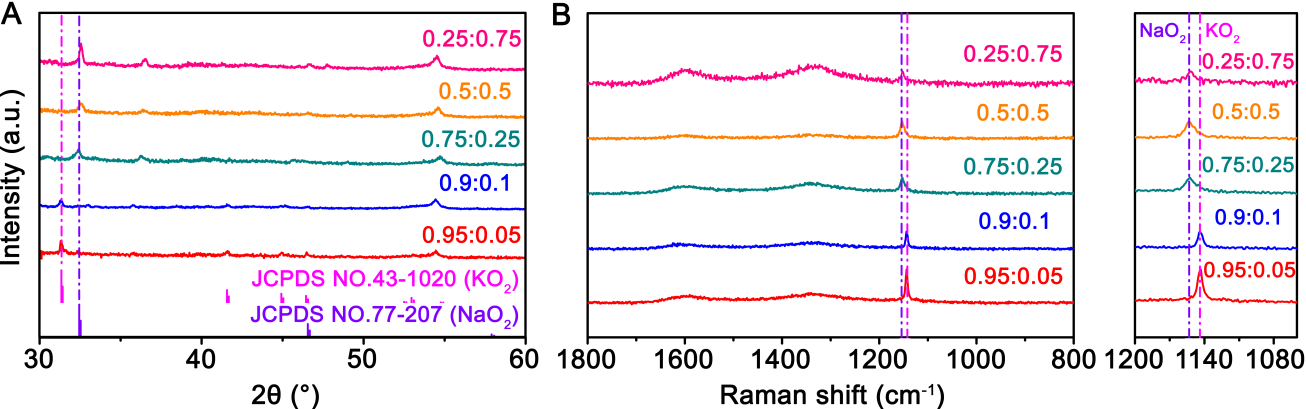


**Fig. S8. Characterization of the discharged SP cathode of the NKO battery.** (A) XRD patterns. (B) Raman spectra. The electrolyte possesses different ratios of [K^+^]:[Na^+^], as indicated. Raman bands of NaO_2_ and KO_2_ are located at 1156 and 1142 cm^-1^, respectively. Discharge capacity: 1000 mAh g^-1^; current density: 500 mA g^-1^; cathode loading: 0.4 mg cm^-2^.

**
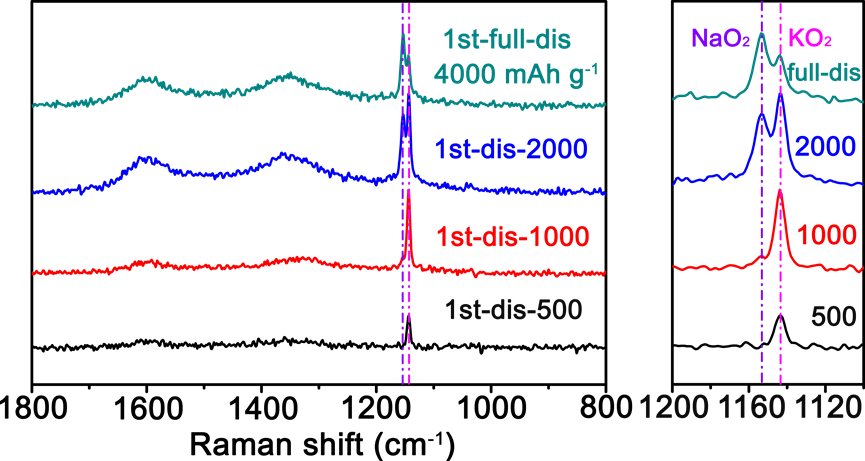
**

**Fig. S9. Raman spectra of the discharged SP cathodes in the NKO battery at different discharge depths.** It indicates that the NaO_2_ appears in the cathode with the increase of the discharge capacity of the NKO battery from 500 and 1000 mAh g^-1^ to 2000 and 4000 mAh g^-1^. Raman bands: 1142 cm^-1^ (NaO_2_), 1156 cm^-1^ (KO_2_); Current density: 500 mA g^-1^; cathode loading: 0.4 mg cm^-2^.

**
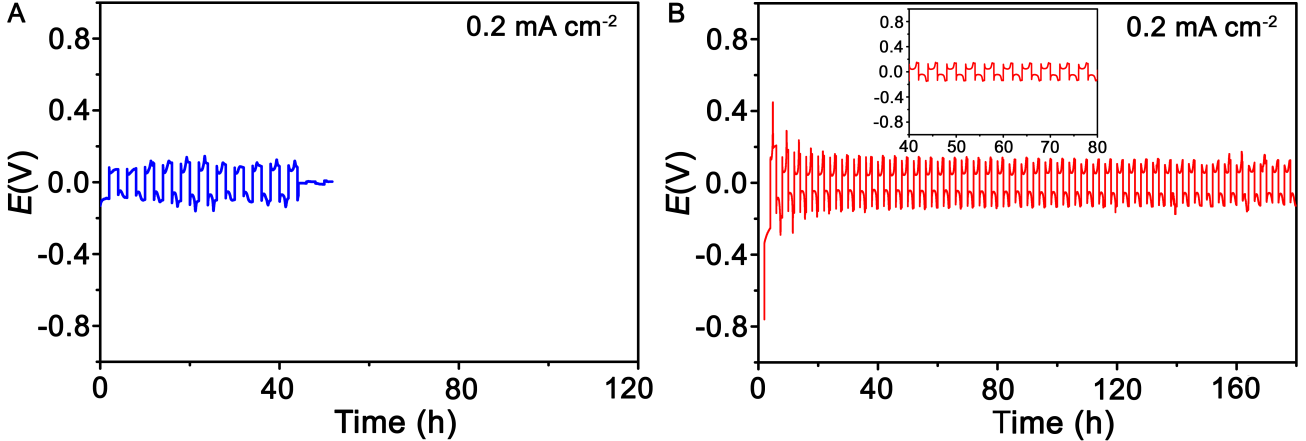
**

**Fig. S10.** Plots of voltage versus time of symmetric Na/Na cells with 1.0 M NaOTF (A) and KOTF (B) in G2, respectively, at 0.1 mA cm^-2^ in O_2_ atmosphere. The inset is the magnified curve.

**
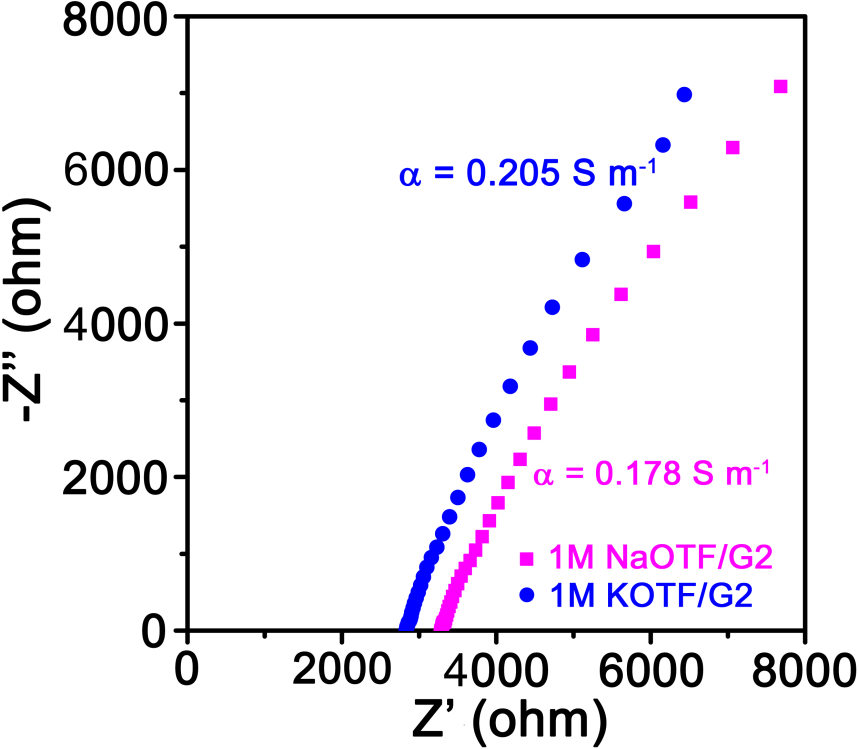
**

**Fig. S11. Electrochemical impedance spectroscopy of the two kinds of electrolytes, 1.0 M NaOTF and 1.0 M KOTF in G2.** From the spectra, the ionic conductivity of 1.0 M KOTF in G2 (blue) is higher than that of 1.0 M NaOTF in G2 (magenta). α = 1 / ρ (α, ionic conductivity; ρ, resistance).


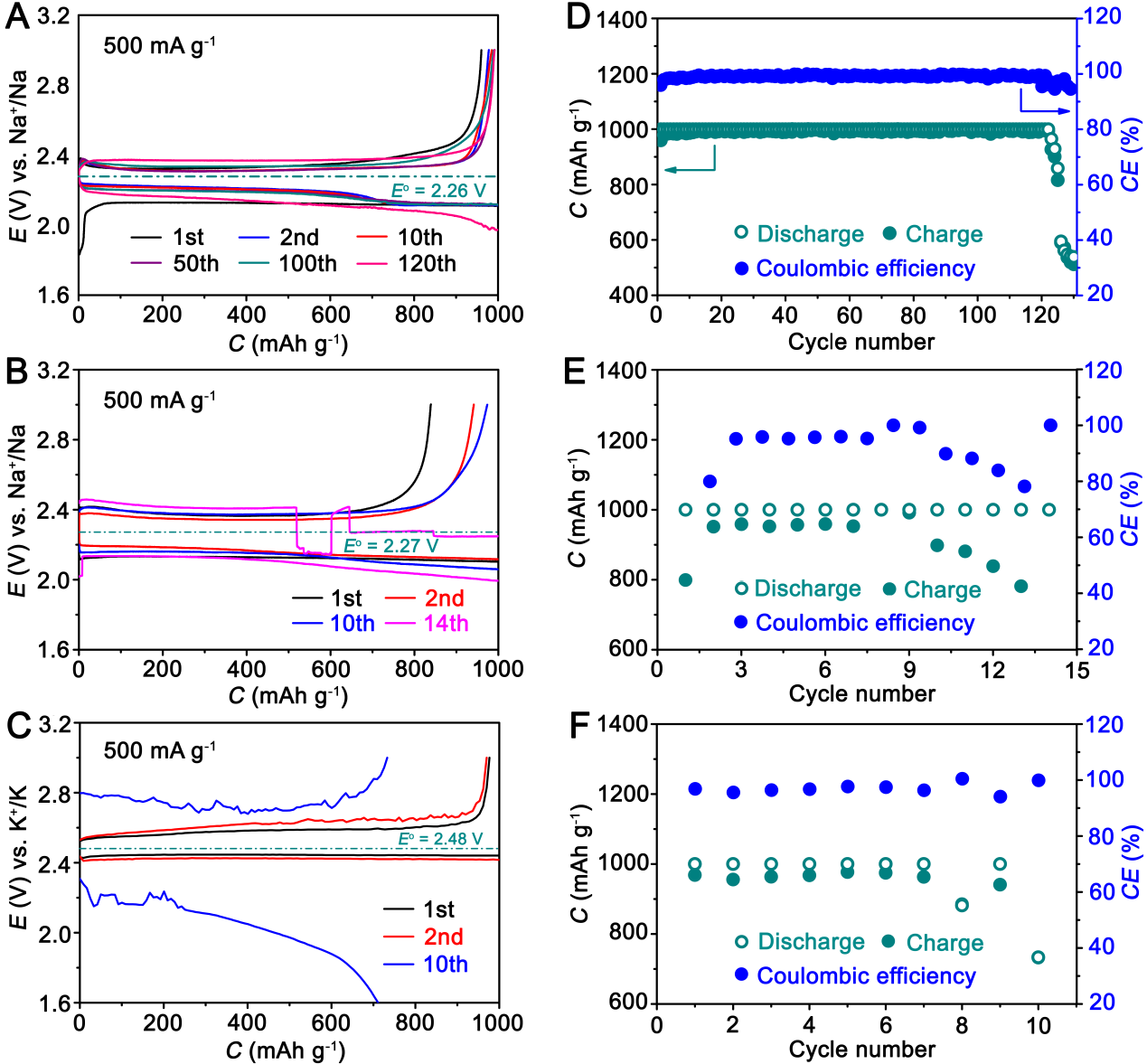


**Fig. S12. Electrochemical performances of NKO, Na-O_2_, and K-O_2_ battery.** (A) NKO. (B) Na-O_2_. (C) K-O_2_ battery and the corresponding cycling performance (D, E, F) at 500 mA g^-1^ with a capacity limit of 1000 mAh g^-1^. The cathode is carbon paper coated with SP. Cathode loading: 0.4 mg cm^-2^. The applied electrolytes are 1.0 M KOTF (A, C) and 1.0 M NaOTF (B) in G2, respectively.

**
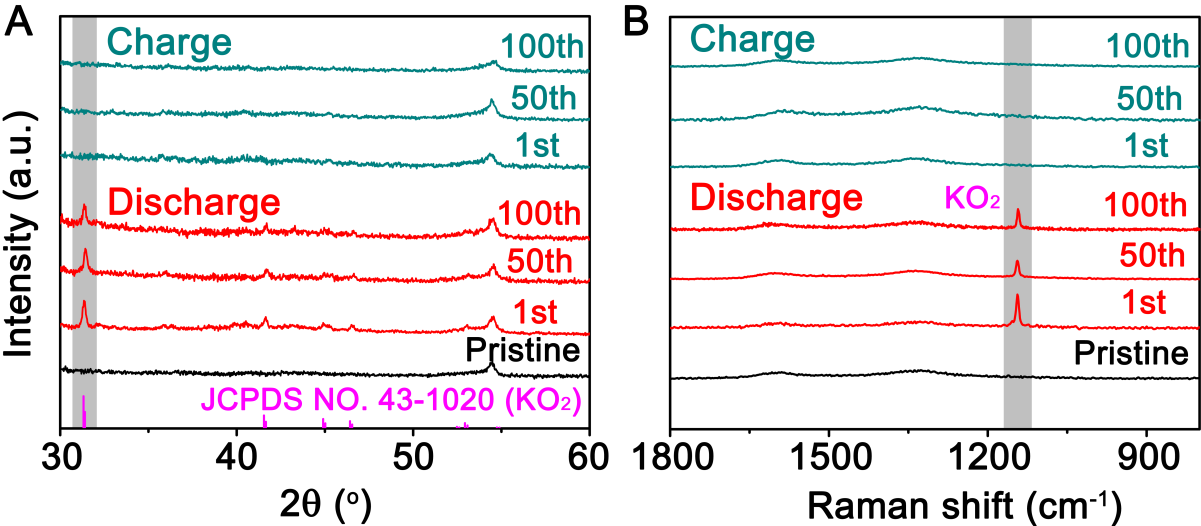
**

**Fig. S13. Analyses on the discharged/charged SP cathodes of the NKO battery during cycles.** (A) XRD patterns. (B) Raman spectra.


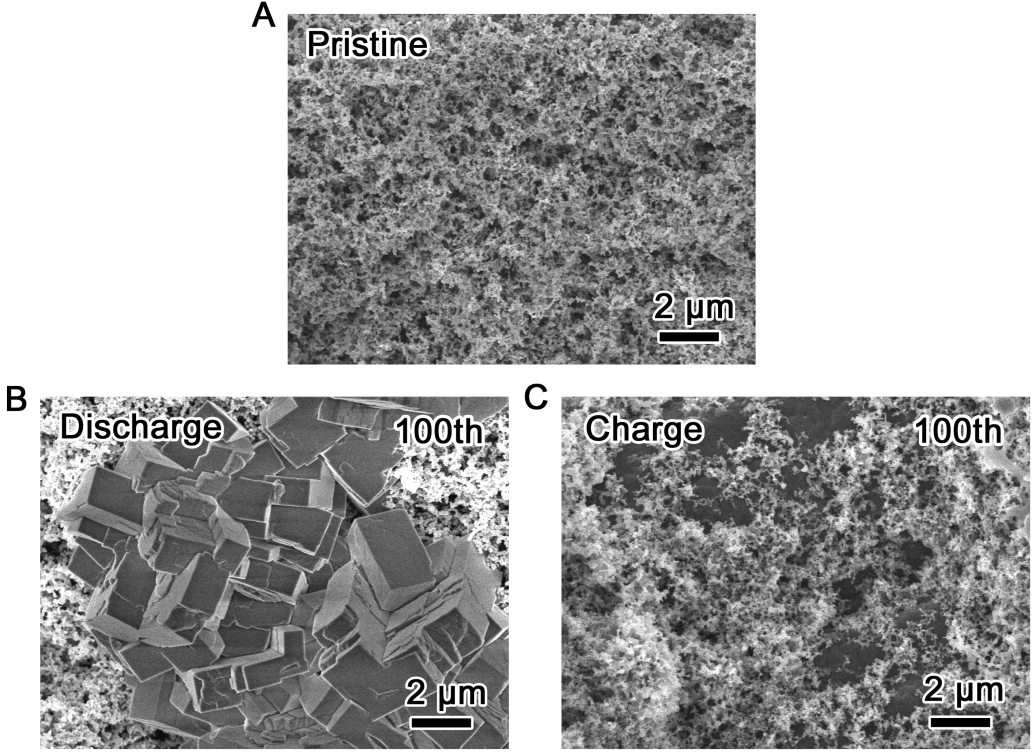


**Fig. S14. SEM images of the pristine and discharged/charged SP cathodes of the NKO battery.** (A) Pristine SP cathode. (B) Discharged SP cathode. (C) Charged SP cathode.


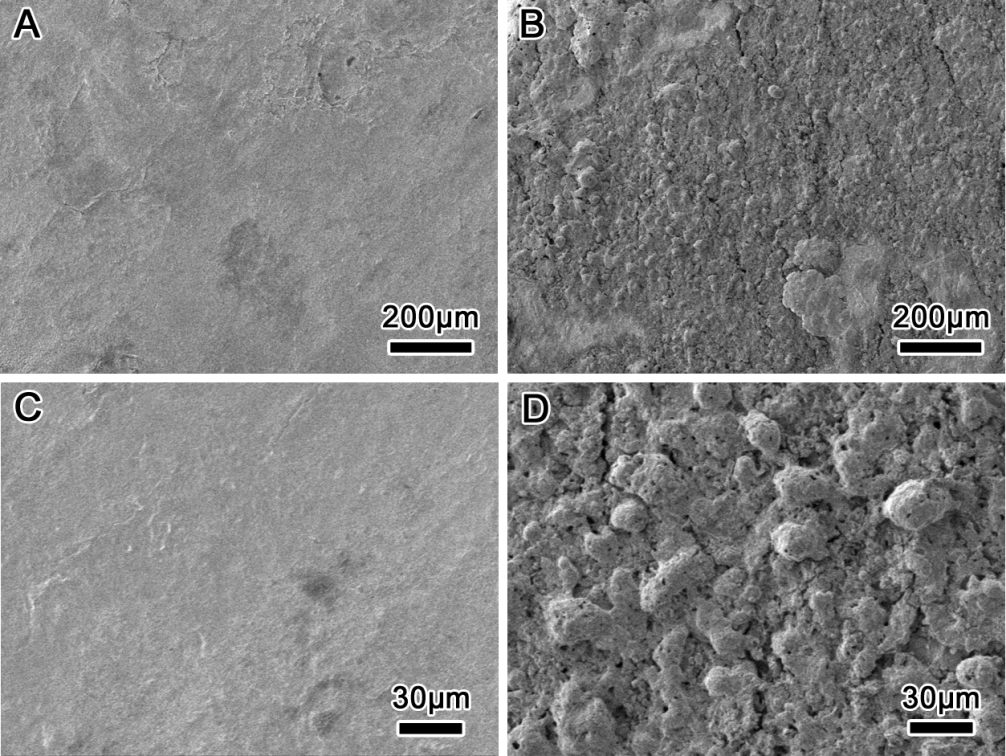


**Fig. S15. SEM images of Na anodes.** (A,C) Pristine Na. (B,D) Na of Na-O_2_ battery in the tenth cycle.

**Table S1. Comparison of NaO_2_ and KO_2_.** KO_2_ is more stable and has higher conductivity than NaO_2_.

|  | NaO_2_ | KO_2_ |
| --- | --- | --- |
| Syngony | Cubic | Tetragonal |
| Space group | *Fm-3m* | *I4/mmm* |
| Stability | NaO_2_→Na_2_O_2_•2H_2_O Unstable | Thermodynamically  stable |
| Conductivity | 4 × 10^-17^ S cm^-1^ (*15*) | 50 S cm^-1^ (*13, 44*) |

**Supplementary Methods**

**Iodometric** **titration**

**(i) Preparation of standard sodium thiosulfate (Na_2_SO_3_) aqueous solution**

0.001 M of Na_2_SO_3_ aqueous solution is prepared by dissolving 0.0625 g of Na_2_SO_3_ · 5H_2_O and 0.05 g of sodium carbonate (Na_2_CO_3_) in 250 mL of distilled water. The concentration of Na_2_SO_3_ solution is calibrated according to the equation of (1) and (2). Firstly, 1.5 mg of K_2_Cr_2_O_7_ was weighed. It was then added into 2.5 mL of an aqueous solution containing 20 mg of KI to generate quantitative I_2_. It was diluted to 2.5 mM, and was used to titrate the prepared Na_2_SO_3_ solution. Finally, the concentration of Na_2_SO_3_ was calibrated to be 1.1 mM.

Involved reactions:

Cr_2_O_7_^2-^ + 6I^-^ + 14H^+^ → 2Cr^3+^ + 3I_2_ + 7H_2_O (1)

2S_2_O_3_^2-^ + I_2_ → S_4_O_6_^2-^ + 2I^-^ (2)

Stoichiometric relationship:

Cr_2_O_7_^2-^ **～** 3I_2_ **～** 6S_2_O_3_^2-^

**(ii) Titration of KO_2_ in a discharged cathode**

A discharged SP cathode was collected from a disassembled NKO battery in an argon-filled glovebox. It was taken out and immediately put into 10 mL of water. After no gas bubbles were generated, the solution was transferred into a conical flask, into which 25 mL of buffered solution (6.5 mg of ammonium paramolybdate, 0.11 mol H_2_PO_4_^-^, 0.03 mol HPO_4_^2-^, and 67 g of KI in 100 mL of distilled water) was added. The solution turned to yellow, indicative of I^-^ in the solution oxidized to I_2_ by H_2_O_2_. During titration with the Na_2_S_2_O_3_ solution, it gradually became light color for the reaction between I_2_ and Na_2_S_2_O_3_. When the color of the solution turned into pale yellow, 0.5 mL of starch indicator (5 g L^-1^) was added and the solution changed to blue. The titration was finished till the color disappeared.

Involved reactions:

2KO_2_ + 2H_2_O → 2KOH + H_2_O_2_ + O_2_ (3)

H_2_O_2_ + 3I^-^ + 2H^+^ ↔ 2H_2_O + I_3_^-^ (4)

I_3_^-^ + 2S_2_O_3_^2-^ → S_4_O_6_^2-^ + 2I^-^ (5)

Stoichiometric relationship:

2KO_2_ **～** I_3_^-^ **～** 2S_2_O_3_^2-^

**Titrations conducted on discharged SP cathodes** (Capacity limit: 0.3 mAh, equal to 11.21 μmol e^-^).

|  | 1 | 2 | 3 |
| --- | --- | --- | --- |
| V (Na_2_S_2_O_3_, mL) | 10.32 | 10.42 | 10.15 |
| KO_2_ (μmol) | 11.33 | 11.44 | 11.11 |
| e^-^/O_2_ | 1.01 | 1.02 | 0.99 |

**Estimation of reactions occurring on the anode and cathode**

The reactions occurring on the anode and cathode and the theoretical potentials are described below:

Anode:

Na^+^ + e^-^ = Na $E_{1}^{\theta}$ = -2.84 V

K^+^ + e^-^ = K $E_{2}^{\theta}$ = -3.08 V

Standard potential of Na^+^/Na (K^+^/K) in G2 was measured in a three-electrode cell, using a Na (K) foil as the working electrode, a Pt plate as the counter electrode, and a silver wire as pseudo-reference electrode. Ferrocenium/ferrocene (Fc^+^/Fc) was used as inner reference to calibrate the pseudo-reference electrode. The applied electrolyte was 1.0 M of NaOTF (KOTF) in G2.

Cathode:

Na^+^ + e^-^ + O_2_ = NaO_2_ $E_{3}^{\theta}$ = -0.57 V

K^+^ + e^-^ + O_2_ = KO_2_ $E_{4}^{\theta}$ = -0.60 V

Nernst equation: $E=E^{\theta}-\frac{RT}{nF}ln\frac{a(O)}{a(R)}$

The critical concentration ratios ([K^+^]/[Na^+^]) on anode and cathode are obtained from the Nernst equations. The detailed calculative processes are showed as follow:

Anode side:

$$E_{Na}=E_{1}^{\theta}-\frac{RT}{F}ln\frac{a(Na)}{a\left( {Na}^{+} \right)}$$

$$E_{K}=E_{2}^{\theta}-\frac{RT}{F}ln\frac{a(K)}{a\left( K^{+} \right)}$$

If $E_{Na}=E_{K}$

$$ln\frac{a(K^{+})}{a({Na}^{+})}=9.34$$

$$\frac{a(K^{+})}{a({Na}^{+})}=11368$$

When $\frac{a(K^{+})}{a({Na}^{+})}>11368$ , K^+^ will be plated onto the anode in a charging process. In the first charge of the NKO battery (CE = 96%) (capacity, 0.3 mAh; electrolyte, 100 μL, assuming the electrolyte loss is 50%), $\frac{a(K^{+})}{a({Na}^{+})}=112.64\ll11368$. Therefore, only Na^+^ is plated onto the Na anode, leaving K^+^ in the electrolyte.

Cathode side:

$$E_{NaO2}^{'}=E_{3}^{\theta}-\frac{RT}{F}ln\frac{a(NaO_{2})}{a\left( {Na}^{+} \right)p(O_{2})}$$

$$E_{KO2}^{'}=E_{4}^{\theta}-\frac{RT}{F}ln\frac{a(KO_{2})}{a\left( K^{+} \right)p(O_{2})}$$

If $E_{NaO2}^{'}=E_{KO2}^{'}$

$$ln\frac{a(K^{+})}{a({Na}^{+})}=1.167$$

$$\frac{a(K^{+})}{a({Na}^{+})}=3.213$$

When $\frac{a(K^{+})}{a({Na}^{+})}<3.213$, Na^+^ will combine with superoxide to form NaO_2_. After the the first discharge of the NKO battery, there coexist K^+^ and Na^+^ in the electrolyte, the ratio between which is $\frac{a(K^{+})}{a({Na}^{+})}=3.45>3.213$. Therefore, the discharge product is only KO_2_. When the limited discharge capacity is 2000 mAh g^-1^ (0.6 mAh) or 4000 mAh g^-1^ (1.2 mAh), the ratio of the remaining K^+^ and Na^+^ is $\frac{a(K^{+})}{a({Na}^{+})}=1.27<3.213$ or$\frac{a(K^{+})}{a({Na}^{+})}=0.14<3.213$, then NaO_2_ is generated together with KO_2_ in the cathode. It should be noted that these calculations are performed without consideration of kinetics, namely, effect of currents, which usually induce large polarization. In this manuscript, the applied current density is not high enough to alter the sequence of deposition of Na and K, and formation of KO_2_ and NaO_2_ as presented above.

**Calculation of theoretical equilibrium potential**

The reactions of the NKO battery are shown as follow:

Anode: Na ‒ e^-^ → Na^+^

Cathode: K^+^ + e^-^ + O_2_ → KO_2_

Total: Na + K^+^ + O_2_ → Na^+^ + KO_2_

Na^+^ + e^-^ → Na ${\Delta G}_{1}^{\theta}$ = 274.02 kJ mol^-1^

K^+^ + e^-^ → K ${\Delta G}_{2}^{\theta}$ = 297.17 kJ mol^-1^

K + O_2_ → KO_2_ ${\Delta G}_{3}^{\theta}$ = -239.40 kJ mol^-1^

Theoretical equilibrium potential ($E^{\theta}$) of the NKO battery depends on Gibbs free energy difference (${\Delta G}^{\theta}$) listed above:

${\Delta G}^{\theta}={\Delta G}_{3}^{\theta}-{\Delta G}_{1}^{\theta}+{\Delta G}_{2}^{\theta}$ = -218.15 kJ mol^-1^

$E^{\theta}=-\frac{{\Delta G}^{\theta}}{nF}$ = 2.26 V
